# Virtual Reality Gamification of Visual Search, Response Inhibition, and Visual Short-Term Memory Tasks for Cognitive Assessment: Experimental Study

**DOI:** 10.2196/65836

**Published:** 2025-07-29

**Authors:** Marios Hadjiaros, Andria Shimi, Kleanthis Neokleous, Constantinos Pattichis, Marios Avraamides

**Affiliations:** 1 CYENS—Centre of Excellence, 1 Dimarchou Lellou Demetriadi, Nicosia, 1016, Cyprus, 357 99094970; 2 Department of Computer Science, University of Cyprus, Nicosia, 1678, Cyprus; 3 Biomedical Engineering Research Centre, University of Cyprus, Nicosia, Cyprus; 4 Department of Psychology, University of Cyprus, Nicosia, Cyprus

**Keywords:** attention, memory, virtual reality, visuospatial, serious games, cognitive assessment, Corsi Test, Visual Search, Go/No-Go, response inhibition

## Abstract

**Background:**

Cognitive tasks are foundational tools in psychology and neuroscience for studying attention, perception, and memory. However, they typically employ simple or artificial stimuli and require numerous repetitive trials, which can adversely affect participant engagement and ecological validity.

**Objective:**

This study investigated whether gamified versions of 3 established cognitive tasks, namely, the Visual Search task (attention), the Whack-the-Mole task (response inhibition), and the Corsi block-tapping test (visual short-term memory), replicate the typical patterns of results reported for their traditional counterparts. It also examined whether the method of administration—in immersive virtual reality (VR) versus desktop computer, and in the laboratory versus at home—influences performance.

**Methods:**

Seventy-five participants (male=24, female=51; age range 18‐35 years; mean 23.15, SD 4.38 years) were randomly assigned to 1 of 3 administration conditions (n=25 each). In the VR-Lab condition, participants completed the tasks in immersive VR within the laboratory; in the Desktop-Lab condition, they completed the tasks on a 2D desktop screen in the laboratory; and in the Desktop-Remote condition, participants completed the tasks on their personal computers at home. All participants completed the same gamified tasks while seated, entering responses with either a mouse or a VR controller, depending on the condition.

**Results:**

The results obtained from these gamified tasks across all 3 administration conditions replicated the typical performance patterns observed with their traditional counterparts, despite using more ecologically valid stimuli and fewer trials. However, administration modality did influence certain performance measures, particularly reaction times (RTs) and task efficiency. Specifically, in the Visual Search task, RTs were significantly faster in the VR-Lab condition (mean 1.24 seconds) than in the Desktop-Lab (mean 1.49 seconds; *P*<.001) and Desktop-Remote (mean 1.44 seconds; *P*=.008) conditions. In the Whack-the-Mole task, no significant group differences emerged in d’ scores (VR-Lab: mean 3.79, Desktop-Remote: mean 3.75, Desktop-Lab: mean 3.62; *P*=.49), but RTs were slower in the Desktop-Remote condition (mean 0.64 seconds) than in the VR-Lab (mean 0.41 seconds; *P*<.001) and Desktop-Lab (mean 0.48 seconds; *P*<.001) conditions. For the Corsi block-tapping test, no significant group differences in span scores were found (VR-Lab: mean 5.48, Desktop-Lab: mean 5.68, and Desktop-Remote: mean 5.24; *P*=.24). Finally, a significant positive correlation was observed between RTs for Hits in the Whack-the-Mole task and feature search trials in the Visual Search task (*r*=0.24; *P*=.04).

**Conclusions:**

Gamified cognitive tasks administered in VR replicated established behavioral patterns observed with their traditional versions while improving ecological validity and reducing task duration. Administration modality had limited effects on overall outcomes, although RTs were slower in remote settings. These findings support the feasibility of using gamified VR tasks for scalable and ecologically valid cognitive assessment. Overall, the study underscores the potential of VR to increase participant engagement and enrich cognitive research through more immersive and motivating testing environments.

## Introduction

### Background

Cognitive tasks are commonly used in Psychology and Neuroscience to investigate various mental processes, such as attention, memory, and perception [1,2]. These tasks often involve simple stimuli, such as alphanumeric characters and shapes and a large number of trials, a deliberate design choice aimed at controlling for extraneous variables and ensuring the reliability of the results [3,4]. While this methodological approach is crucial for maintaining experimental rigor and validity, it may inadvertently lead to issues regarding participant engagement [5]. For example, the repetitive tasks and the simple stimuli may cause participant disinterest, boredom, and fatigue, which in turn may compromise data quality or introduce unintended biases [6,7]. Thus, while striving for methodological control, researchers must also consider strategies to enhance participant engagement to mitigate these potential challenges and maintain the integrity of their findings.

### Virtual Reality as a Tool for Gamified Cognitive Assessment

Virtual reality (VR) technology presents a promising avenue for addressing the engagement challenges inherent in traditional cognitive tasks [8]. By leveraging VR, researchers can gamify tasks, transforming them into immersive and interactive experiences that are inherently more engaging for participants [9].

Numerous studies have indicated that gamification in cognitive tasks can significantly increase participant engagement and motivation [10,11]. Recent studies exploring VR gaming have emphasized the significant role of user engagement and motivation in enhancing the overall experience. One key factor driving VR game adoption is the concept of “flow,” which is a state of optimal immersion where players experience intense enjoyment and concentration. This “flow” state is essential for fostering deeper engagement with VR games, particularly as the player becomes more immersed in the virtual environment. The sense of spatial presence, or the feeling of being physically located within the VR world, is another major motivator, as it strengthens the emotional connection to the game and enhances relaxation or enjoyment [12,13].

### Cognitive Tasks Under Investigation

The Visual Search task is a widely used method for investigating the mechanisms of attention involved in locating a target among distractors, mirroring real-world scenarios such as finding a friend in a crowded cafeteria, searching for an item in the grocery store, or searching a target object among nontarget objects during security screening. In laboratory settings, a typical Visual Search task involves presenting a target object on the computer screen among distractors [14,15]. Stimuli often consist of alphanumeric characters or simple geometric shapes. Visual search tasks typically involve 2 key manipulations: similarity and display size. Similarity refers to the type of search that is determined by how visually distinct the target is from the distractors. In *feature search* trials, the target differs from distractors in terms of a single feature, such as its shape (eg, locating the letter “O” among several “Xs”) or its color (eg, locating a red “X” among white “Xs”). In *conjunction search* trials, the target is defined by a combination of features that are also present in the distractors (eg, locating a green “O” among green “Xs” and red “Os”). The second manipulation is the display size, which refers to the number of distractors presented in each trial alongside the target [16]. The typical pattern of results obtained across many studies is that (1) reaction times (RTs) (ie, the time it takes the participant to find the target from the onset of the display) are shorter for feature search trials than conjunction search trials, (2) RT increases significantly with display size, a phenomenon known as the “display size effect,” but only in conjunction search trials [15,17,18], and (3) RT remains relatively constant across display sizes in feature search trials, a phenomenon known as the “pop-out effect” [19].

The Whack-the-Mole task is a Go/No-Go task that provides a measure of response inhibition, that is, the ability to inhibit inappropriate prepotent responses [20]. Past research has shown that response inhibition is a key ability for cognitive [21] and psychosocial [22] development and is a reliable predictor of school readiness and academic achievement in domains such as math and reading [23]. Impairments in response inhibition are commonly observed in neurodevelopmental disorders such as autism [24] and attention-deficit/hyperactivity disorder [25,26] as well as in psychiatric and neurological disorders such as borderline personality disorder [27] and Parkinson disease [28]. In computerized Go/No-Go tasks, participants are required to execute an action rapidly (eg, press a button) upon detecting a specific stimulus (Go trials) and withhold a response when presented with a different stimulus (No-Go trials) [20,21]. To ensure task effectiveness, the majority of trials are typically Go trials (approximately 75%), priming the execution of a response [20,21]. Response inhibition is assessed by computing d’, a sensitivity metric that takes into account the proportion of hits (ie, correctly executed responses in Go trials) and false alarms (ie, incorrect execution of responses in No-Go trials) [20,29]. Although d’ may vary depending on the manipulations of the experiment, such as the discriminability of the stimuli, its typical value, indicating moderate sensitivity, is between 3 and 4 [20,30]. In general, participants exhibit false alarms in 10% of the No-Go trials [20,21].

The Corsi Test is one of the most widely used tasks to assess visuospatial short-term memory. The original version [31] consists of 9 physical cubes placed on a board. Each trial involves the experimenter sequentially touching a series of blocks from one side of the board, and the participant, situated on the opposite side, is required to reproduce the sequence by touching the blocks in the same order. If the participant successfully reproduces the sequence, then the number of blocks touched in the sequence is increased by 1 [31-33]. The task continues until the participant fails to accurately reproduce the sequence [34]. The longest sequence that is reproduced correctly indexes the participant’s visuospatial short-term memory span. Studies show that healthy adults have a typical span between 5 and 7 [35]. In more recent implementations, electronic versions of the task have been developed and delivered through computers or mobile devices, where targets light up in a specific sequence that participants reproduce by clicking or touching the targets. Even in these modern versions of the task, the typical span ranges between 5 and 7 [33].

### Related Work

Immersive VR limits extraneous distractions from the surrounding environment [36,37]. This is because immersive VR setups entail wearing a head-mounted display (HMD) that effectively blocks out the external environment, thereby reducing potential environmental distractions that may interfere with task performance [38,39]. This feature may be particularly important in cognitive research, where maintaining participants’ focus and attention is being assessed [40] and a measure of “true” ability under no external distraction is needed. It is, therefore, imperative to ascertain whether these gamified tasks, administered with or without immersive VR, produce the expected pattern of results as their traditional counterparts, an indication that they capture the same underlying cognitive processes. Recent studies have investigated the consistency and relative performance of computerized and VR-based cognitive tasks, revealing nuanced differences between the 2 modalities. These findings underscore the importance of task context and cognitive demands when evaluating their efficacy.

For example, Barrett et al [41] compared VR, 3D desktop, and 2D versions of a category learning task in which participants classify visual stimuli into categories based on 2 relevant features while ignoring a third irrelevant one. Feedback is provided after each trial to support learning. RTs in the 2D condition were significantly faster than in the VR and 3D conditions, and fixation durations were shorter, indicating reduced cognitive load for the 2D environment. However, participants reported higher levels of immersion and engagement in the VR and 3D conditions, suggesting that while traditional computerized tasks may facilitate quicker responses, VR environments offer richer experiential benefits.

This trade-off between speed and immersion is also reflected in findings from Bhargava et al [42], who examined cognitive task performance across VR and mobile-based conditions using custom-designed games. Bhargava et al [42] validated 3 custom-designed games: 2 VR-based (Navigation and Hand-Eye Coordination) and 1 tablet-based (Memory) against the ACE-III (Addenbrooke’s Cognitive Examination—III) cognitive assessment tool in a cohort of young adults. The ACE-III is a comprehensive tool used to assess cognitive functioning across 5 domains: attention, memory, language, verbal fluency, and visuospatial skills. The study found no significant differences in overall task performance between the VR and 3D mobile conditions; however, the VR tasks elicited significantly higher motivation and engagement scores. These findings suggest that VR can enhance user experience and engagement without compromising the validity or reliability of cognitive assessment.

This enhanced motivational effect of VR-based tasks is further reinforced by Faria et al [43], who explored VR in the context of cognitive rehabilitation. Similar findings are reported by Faria et al [43], who evaluated 2 approaches to personalized cognitive rehabilitation—1 VR-based and 1 traditional—with patients with chronic stroke. Both methods were equally effective in improving cognitive performance, but the VR approach led to higher participant satisfaction and engagement due to its interactive features. This suggests that VR can deliver comparable therapeutic outcomes with added motivational benefits.

In addition to motivation, several studies emphasize the ecological validity of VR, a point illustrated well in the work by Tan et al [44]. Tan et al [44] assessed a VR-based cognitive screening tool targeting 6 cognitive domains. Their results showed high accuracy in detecting cognitive impairments, comparable with traditional methods, with the added benefit of enhanced ecological validity in VR. Participants were able to perform naturalistic tasks, such as navigation and object interaction, which are difficult to replicate in conventional computerized tests.

This growing interest in ecological validity and realism of cognitive assessments in VR is echoed in the meta-analysis by Neguţ et al [45], which further consolidates the strengths and challenges of immersive assessments. Finally, Neguţ et al [45] conducted a meta-analysis comparing VR-based assessments with classical paper-and-pencil or computerized measures. They reported that VR tasks often felt more challenging due to their immersive and dynamic nature, which can increase perceived cognitive load. However, the ecological validity and potential for more realistic stimuli in VR environments often make them more representative of real-world cognitive challenges.

In summary, past research shows that VR-based tasks enhance immersion, engagement, and ecological validity, making them a compelling alternative to traditional assessments. While RTs may vary depending on task design and context, this trade-off is often outweighed by the benefits of more naturalistic and interactive environments. These advantages position VR as a valuable complement to conventional methods, particularly in contexts that require high levels of interaction, naturalistic task performance, or strong participant motivation.

Building on this body of work, this study extends previous research by directly comparing attention and memory performance in immersive VR with desktop settings, both in-lab and remote, to assess the impact of presentation medium and testing environment on cognitive outcomes. Related to this question, several past studies using a variety of tasks have indicated that the quality of data collected on the web remains uncompromised [46-52]. However, web-based data frequently exhibit greater variability in contrast to data gathered in controlled laboratory environments [51]. This variability could either stem from the diminished control over experimental conditions, as previously mentioned, or it could be an intrinsic characteristic of studying more diverse participant cohorts, thereby becoming a variable of interest in its own right [53].

This issue of variability is particularly relevant in light of findings by Segen et al [54], whose online versus lab-based comparison provides an important context for interpreting remote testing results. Notably, Segen et al [54] found that the data collected in a conventional laboratory setting and those collected on the web produced very similar results, although the web-based data were more variable, with SEs being about 10% larger than those of the data collected in the lab. However, Segen et al [54] have used a scene recognition task that might be less prone to the environmental distraction that is typically present when carrying out a task remotely compared with the attention and memory tasks we used here.

### Problem Statement and Objective

The heightened engagement resulting from the use of VR may come at a cost to experimental control. The dynamic and immersive nature of VR environments introduces additional variables that may influence participants’ cognitive processes, potentially altering the patterns of results obtained compared with their traditional counterparts. Therefore, it becomes imperative for scientists to systematically evaluate whether gamifying cognitive tasks in VR environments yields findings that are consistent with those obtained with traditional methods [19]. Doing so would ensure that while enhancing engagement with gamification, researchers can still maintain the methodological rigor necessary for robust scientific conclusions. Therefore, the main objective of this study was to examine whether gamified versions of the Visual Search, the Whack-the-Mole, and the Corsi block tasks, each assessing a distinct cognitive process, produce similar patterns of results as their traditional versions. A second objective was to examine whether the way the gamified tasks are administered influences the pattern of results obtained. To this purpose, we compared results across 3 different conditions that involved the same gamified cognitive tasks: an immersive VR condition (VR-Lab), a desktop VR condition (Desktop-Lab; nonimmersive), and a desktop VR condition in which participants carried out the task at home (Desktop-Remote; nonimmersive). By conducting this comparison, we aimed to elucidate the influence of immersive VR on task outcomes, providing insights into the effectiveness of different task formats.

## Methods

### Participants

The experimental study included 75 healthy young adults (male=24, female=51) with an overall mean age of 23.15 years (SD 4.38 years) recruited from the student community of the University of Cyprus and the CYENS—Centre of Excellence. Recruitment was conducted using a convenience sampling method through email announcements, web-based student forums and platforms, and direct invitations. All participants met the following inclusion criteria: (1) aged between 18 and 35 years, (2) normal or corrected-to-normal vision, and (3) no reported history of neurological, psychiatric, or cognitive impairments that could affect task performance. Eligibility was determined through a brief prescreening interview. Individuals with a self-reported history of neurological disorders (eg, epilepsy and traumatic brain injury), psychiatric conditions (eg, attention-deficit/hyperactivity disorder, anxiety, and depression), or uncorrected visual impairments were excluded from participation. Participants were randomly assigned to 1 of 3 between-subject conditions: VR-Lab, Desktop-Lab, or Desktop-Remote, with 25 participants in each group. No participants were excluded from the statistical analyses, and there were no dropouts. All laboratory-based sessions (VR-Lab and Desktop-Lab conditions) took place at the Experimental Psychology Laboratory at the University of Cyprus, a controlled environment designed to minimize external distractions.

### Materials

#### Visual Search Task

**Figure 1.**
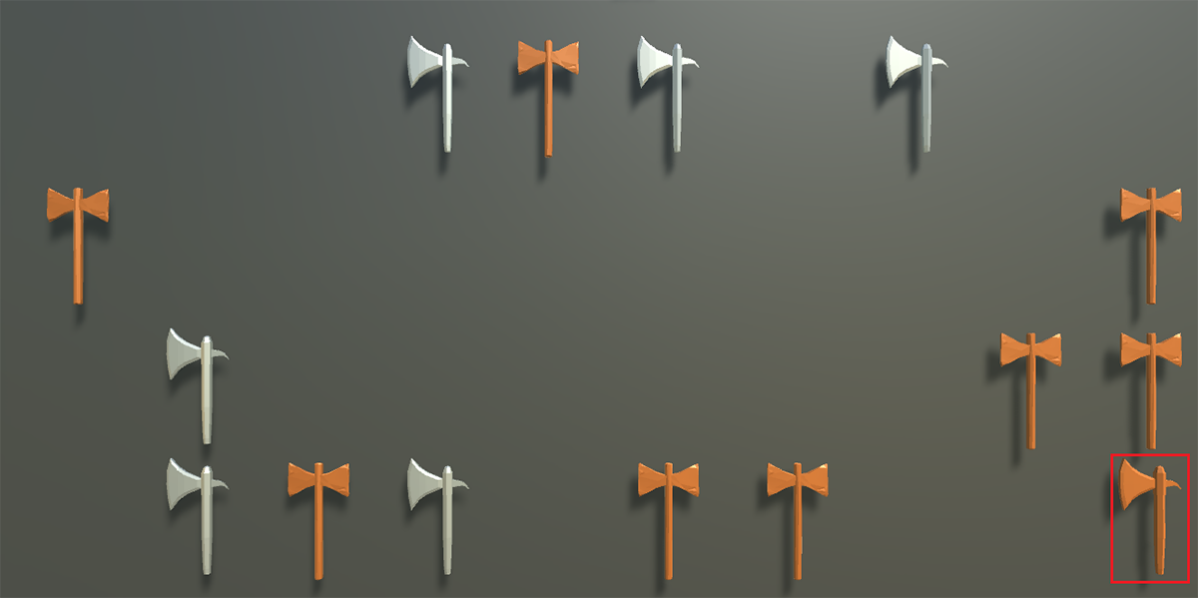
Example of a conjunction search trial in the Visual Search task of the experimental study. In this trial, participants searched for a brown 1-sided axe (target) among gray 1-sided axes and brown double-sided axes (distractors). This is a trial with a display size of 15. The red outline is added for illustration purposes and was not present in the actual display viewed by participants.

The Visual Search task used in this study involved the presentation of brown and gray axes (Multimedia Appendix 1), with the target object changing from trial to trial. In feature search trials, the target differed from the distractors in terms of color, for example, a gray axe presented among brown axes. In contrast, in conjunction search trials, the target was defined by a combination of features that were also present in the distractors. For example, the target could be a brown 1-sided axe presented among gray 1-sided axes and brown double-sided axes (Figure 1). To examine the influence of display size, we varied the number of axes in both trial types to 5, 15, 25, or 40. Participants were instructed to search the display for the target and provide a response as soon as they detected it. In every trial, the targets were presented for 1.5 seconds, followed by the search display. Overall, the task consisted of 96 trials (50% feature search trials and 50% conjunction search trials), with 8 trials in each possible combination of search type and display size. Before the experimental trials, participants performed 8 practice trials, 1 in each combination of search type (Feature vs Conjunction Search) and display size (5, 15, 25, or 40) to familiarize themselves with the task and procedure. Trials were presented in a different random order for each participant. Participants in the VR-Lab condition searched for the target and selected it by directing a laser beam extended from the VR controller toward the target. The response was logged when the laser beam was on the target for 2 seconds; however, the RT was measured from the moment the beam first landed on the target. Participants in the Desktop-Lab and Desktop-Remote conditions responded by moving the mouse cursor to the target and clicking on it. In all 3 conditions (VR-Lab, Desktop-Lab, and Desktop-Remote), participants had the whole board with the axes in their field of view and thus did not need to turn their heads to search.

#### Whack-the-Mole Task

**Figure 2.**
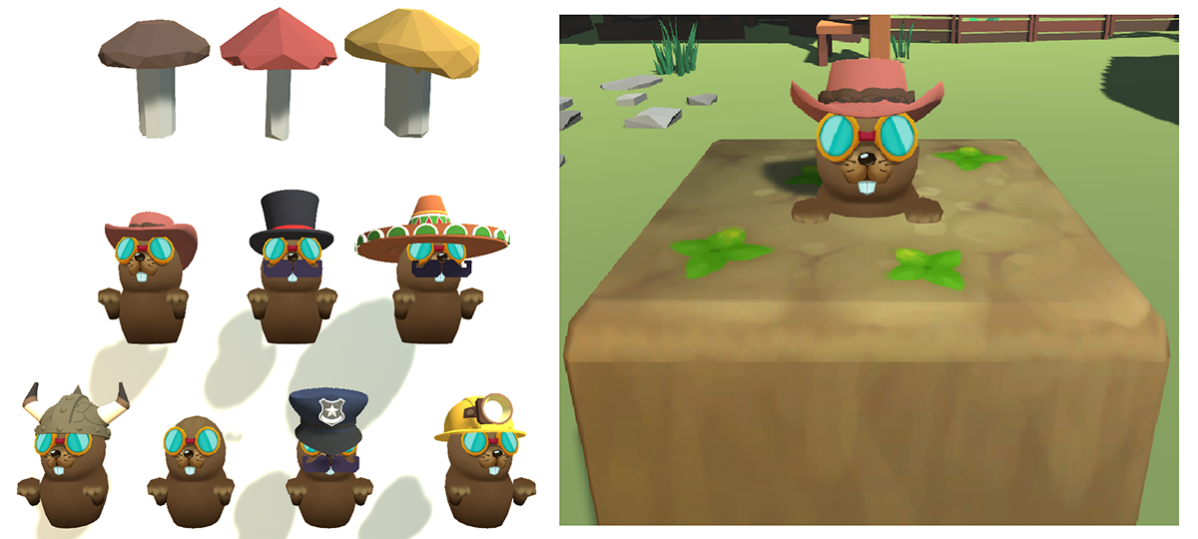
An illustration of the Whack-the-Mole task as used in the study. On the left, the 3 different types of mushrooms (top row) and the 7 different mole disguises (middle and bottom rows) are shown. On the right, an example Go trial is shown. In the VR-Lab condition, participants had to pull the trigger on the handheld controller to respond. In the Desktop-Lab and Desktop-Remote conditions, they had to click the mouse. In No-Go trials, showing different types of mushrooms, participants had to refrain from any response.

In our version of the Whack-the-Mole task that was modeled after Casey et al [55], participants used a mallet to hit moles that popped up with random disguises from the hole in the center of the ground. In Go trials, a mole appeared with a varying disguise in each trial, and participants were instructed to hit it as fast as they could by either pulling the trigger on the handheld controller with the VR-Lab condition or clicking the mouse in the Desktop-Lab and Desktop-Remote conditions. Participants in the VR-Lab condition held a virtual mallet mapped to the controller in their dominant hand and hit the mole by pulling the trigger with their index finger. Similarly, participants in the Desktop-Lab and Desktop-Remote conditions clicked the mouse with the index finger of their dominant hand to hit the mole. In No-Go trials, mushrooms appeared instead of the mole, and participants were instructed to withhold a response (Figure 2). Before the experimental task, participants were shown the 7 disguises of the mole and the 3 types of mushrooms and carried out 10 practice trials. As shown in Figure 2, the task included 7 different mole disguises and 3 different types of mushrooms. Each participant completed 100 trials, with 75 being the Go trials and 25 being the No-Go trials. The 2 trial types were presented randomly within the task. In all 3 conditions (VR-Lab, Desktop-Lab, and Desktop-Remote), participants had a clear view of the ground area from which the mole emerged, without the need to turn their heads.

#### Corsi Test

**Figure 3.**
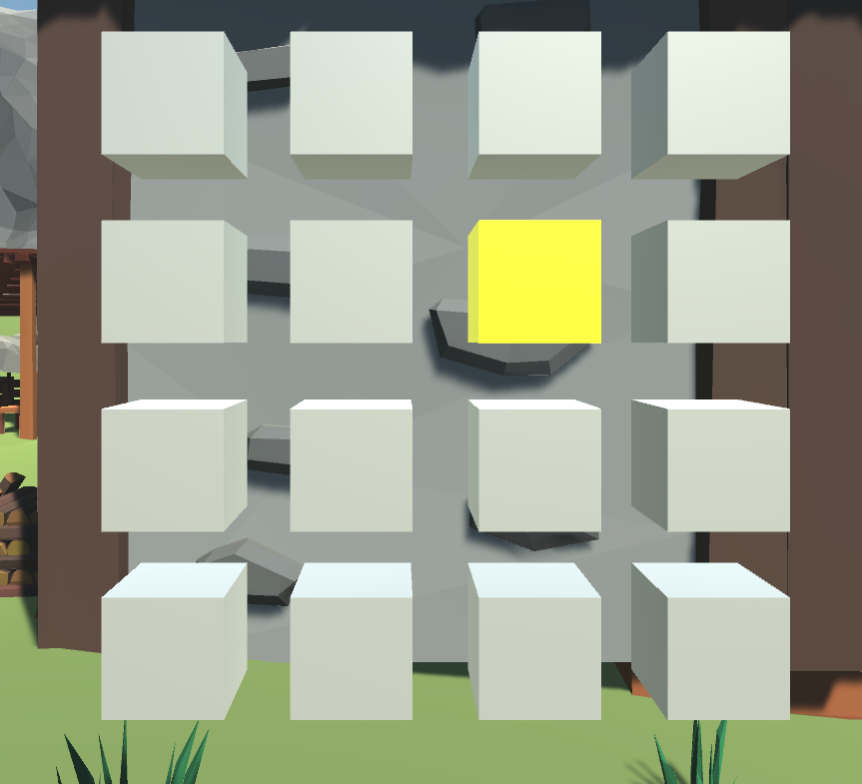
An illustration of the matrix in the Corsi Test as used in this study. Participants viewed a sequence of cubes turning yellow and then recreated it by either touching the cubes with the handheld controllers (VR-Lab condition) or clicking on each cube with the mouse (Desktop-Lab and Desktop-Remote participants).

In this task, participants were presented with a 4×4 matrix of gray cubes (Figure 3). Each trial involved a unique sequence of cubes changing color from gray to yellow sequentially, and participants were required to replicate the sequence by tapping the blocks that changed color in the same order. Participants in the VR-Lab condition used the VR controllers to touch the blocks, whereas those in the Desktop-Lab and Desktop-Remote conditions clicked the blocks with the mouse. The task began with a single cube and the length of the sequence increased as participants responded correctly. Participants were given 3 attempts at each span length and advanced to the next length if they responded correctly in at least 2 attempts. The block span for each participant was measured as the length of the last sequence that was repeated correctly in at least 2 out of 3 attempts. After completing the tasks, participants in the Desktop-Remote condition were asked to click on a save button that sent their data to a cloud database. In all 3 conditions (VR-Lab, Desktop-Lab, and Desktop-Remote), the whole 4×4 matrix was within the participants’ field of view, so no head turning was required.

#### Procedure

Participants were initially contacted via email with details about the study and available time slots for participation. Those interested registered electronically by selecting a suitable time slot. Upon arrival at the lab, participants read a detailed description of the study and signed a consent form if they agreed to take part. No participants withdrew from the experimental procedure. Participants in the Desktop-Remote condition followed the same process, with the only difference being that they completed the study from home through a secure hyperlink and communicated with the experimenter (first author) via video call. They completed the tasks on their personal computers, and they were instructed to select a quiet, distraction-free space and to use a desktop or laptop computer with a stable internet connection. During the experiment, all participants were seated and instructed to use only their hand to operate either the mouse or the VR controller, ensuring that movement was limited primarily to the wrist and minor elbow motion. This protocol was applied consistently across all 3 tasks, regardless of condition. Before starting the experiment, participants in all conditions underwent a brief orientation session where they were familiarized with the tasks and response methods.

Participants in the VR-Lab condition carried out the tasks in an HTC Vive Pro HMD and used the Vive handheld controllers to respond. Participants in the Desktop-Lab condition carried out the task on the same desktop computer that was used for the VR-Lab condition but viewed the tasks on a 24" LCD display. The order of the 3 tasks was counterbalanced across conditions using a 3×3 Latin square design. For all tasks, data in the VR-Lab and Desktop-Lab conditions were automatically recorded by the computer running the experiment. In the 2 lab conditions, data were collected locally on the computer of the lab, while in the Desktop-Remote condition, data were encrypted and stored in a secure database.

### Statistical Design

Separate mixed-design ANOVAs were conducted on mean RTs and d’, depending on the cognitive task (see “Results” section for task-specific details). The within-subject variables for each task are detailed in the “Results” section, while the between-subject variable across all tasks was the mode of administration. In addition to the ANOVAs, Pearson correlations were computed between task scores to explore individual differences. All analyses were carried out using the Jamovi software package [56].

### Ethical Considerations

The study conformed to European and national legislation and fundamental ethical principles, including those reflected in the Charter of Fundamental Rights of the European Union, the European Convention on Human Rights and its Supplementary Protocols, and the World Medical Association Declaration of Helsinki. The protocol of the study was given ethics clearance by the Cyprus National Bioethics Committee (approval number: ΕΕΒΚ ΕΠ 2021.01.99). All participants read and signed informed consent forms either in person or digitally. Data were collected and maintained without any participant identification information.

## Results

### Overview

To investigate whether the 3 gamified tasks replicated the pattern of results reported in the literature for their traditional counterparts and to compare performance across modes of administration, we computed inferential statistics separately for each task. We report the findings in the next subsections.

### Visual Search

For Visual Search, we carried out a repeated-measures ANOVA on RT with search type and display size as the within-subject variables and the administration condition as the between-subject variable.

**Figure 4.**
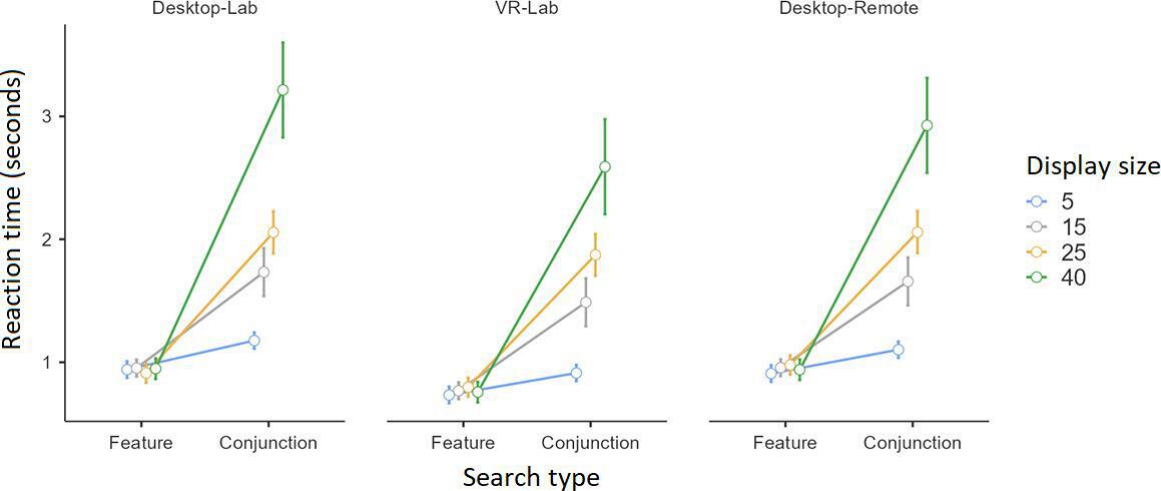
The average reaction time in the Visual Search task as a function of search type (feature search vs conjunction search) and display size (5, 15, 25, and 40 objects) for the VR-Lab, Desktop-Lab, and Desktop-Remote conditions. Results are shown in distinct panels for the VR-Lab, the Desktop-Lab, and the Desktop-Remote conditions. Error bars represent 95% confidence intervals.

**Figure 5.**
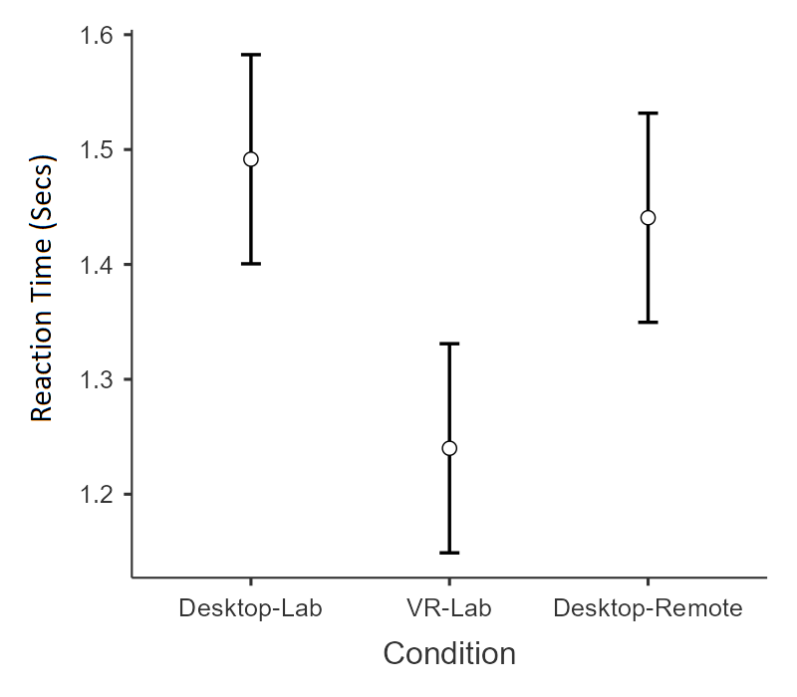
Mean reaction times in the Visual Search task, aggregated across search type and display size, for each of the 3 administration conditions: VR-Lab, Desktop-Lab, and Desktop-Remote. Overall, reaction time was shorter in the VR-Lab condition than in the Desktop-Lab and Desktop-Remote conditions. Error bars represent 95% confidence intervals.

Results revealed significant main effects for search type, *F*_1,72_=787.12, *P*<.001, η^2^=0.38; the display size, *F*_3,216_=150.49, *P*<.001, η^2^=0.17; and the administration condition, *F*_2,72_=8.49, *P*=.001, η^2^=0.02. There was also a significant interaction between search type and display size, *F*_3,216_=145.42, *P*<.001, η^2^=0.16. As seen in Figure 4, the interaction was driven by the presence of a display size effect (ie, RT increasing from 5 to 15 and then to 25 and further to 40 objects) in conjunction search trials but not in feature search trials. The significant effect for the administration condition was due to shorter RT in the VR-Lab (mean 1.24 seconds) condition than in the Desktop-Lab (mean 1.49 seconds; *P*<.001) and Desktop-Remote (mean 1.44 seconds; *P*=.008) conditions (Figure 5). The difference between the Desktop-Lab and Desktop-Remote conditions was not significant (*P*=1.00). Importantly, no significant interaction involving the administration condition was found. The effect size for search type (η^2^=0.38) is large, suggesting a robust difference in RTs between feature and conjunction searches, consistent with prior literature. The effect size for display size (η^2^=0.17) is also large, highlighting the substantial impact of increasing display size on RTs. In contrast, the main effect of the administration condition (η^2^=0.02) has a small effect size, indicating that the mode of task administration had a statistically significant but relatively small influence on RTs. Finally, the interaction effect between search type and display size was associated with a large effect size (η^2^=0.16), reinforcing the well-documented finding that increasing display size disproportionately affects conjunction searches compared with feature searches.

### Whack-the-Mole

To analyze the accuracy data for the Whack-the-Mole task, we first computed for each participant the percentages of hits and false alarms. Based on the percentages, we computed the d’ score for each participant. The d’ score is a sensitive discrimination measure that reflects the degree to which participants accurately report the presence or absence of the target in the display. The d’ score was calculated using the formula: d’=z (hit rate) − z (false alarm rate).

**Figure 6.**
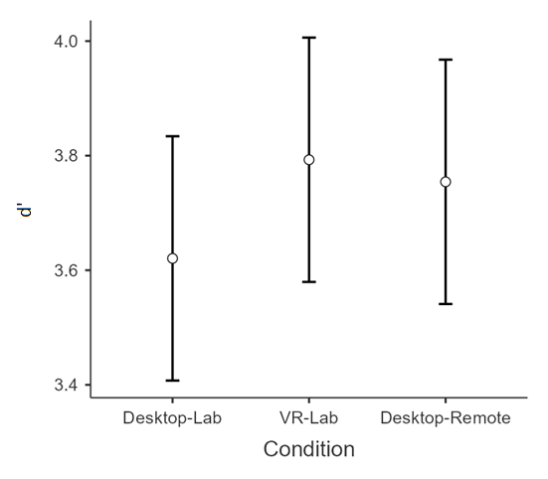
The average d' values in the Whack-the-Mole task across the 3 administration conditions. The d’ is computed, according to Signal Detection Theory, by taking into account the percentage of hits (ie, correct responses to targets) and false alarms (ie, incorrect responses to distractors). Error bars depict 95% confidence intervals.

**Figure 7.**
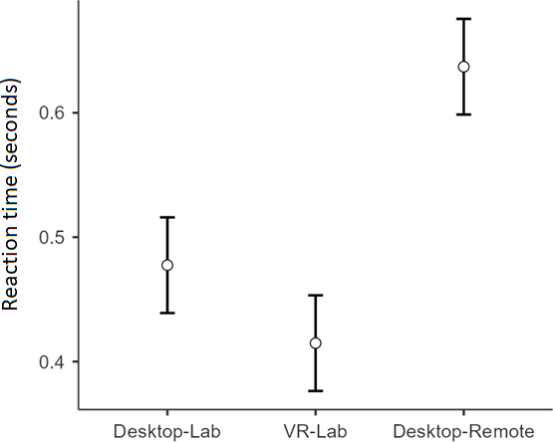
Average reaction time for Hits (ie, correct responses to targets) in the Whack-the-Mole task across the 3 administration conditions. Error bars depict 95% confidence intervals.

The average d’ scores were 3.79 for the VR-Lab, 3.75 for the Desktop-Remote, and 3.62 for the Desktop-Lab condition (Figure 6), in line with the values typically reported in the literature [20,30]. A one-way ANOVA showed that the difference in d’ across the VR-Lab, Desktop-Lab, and Desktop-Remote conditions was not significant, *F*_2,72_=0.71, *P*=.49, η^2^=0.02. In contrast, the analysis on mean RTs for hits only (ie, correct responses in Go trials) showed that participants were significantly slower in the Desktop-Remote condition (mean 0.64 second) than in the Desktop-Lab (mean 0.48 second) and VR-Lab (mean 0.41 second) conditions, both *P* values <.001 (Figure 7). The difference in RTs between the VR-Lab and the Desktop-Lab conditions was marginally significant (*P*=.07).

### Corsi Test

As shown in Multimedia Appendix 2, the average span scores in the Corsi Test were 5.48 in the VR-Lab condition, 5.68 in the Desktop-Lab condition, and 5.24 in the Desktop-Remote condition. Despite the numeric difference across the 3 conditions, a one-way ANOVA showed that the effect of the administration condition on the Corsi span score was not significant, *F*_2,72_=1.45, *P*=.24, η^2^=0.04. These findings indicate that the administration condition had no impact on spatial working memory performance, as measured by the Corsi Test.

### Individual Differences Within Attentional Tasks

**Figure 8.**
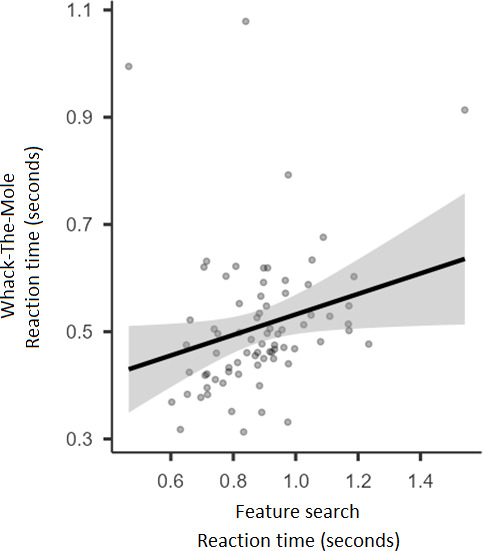
Scatter plot depicting the correlation of Whack-the-Mole reaction time for Hits and Visual Search reaction time for Feature Search trials. The significant positive correlation indicates that participants who were faster to respond in Go trials of the Whack-the-Mole task tended to respond faster in the Feature Search trials of the Visual Search task.

To examine a possible relation between the 2 distinct attentional processes, that is, Visual Search and response inhibition, we carried out Pearson correlation analyses between the mean RT in feature search and conjunction search trials of the Visual Search task separately and the mean RT of Hits in the Whack-the-Mole task. For these, we averaged the RT across displays for each search type and we combined the data from the 3 administration conditions per task. We found a significant positive correlation between RT for Hits in the Whack-the-Mole task and RT in feature search trials of the Visual Search task, *r*_73_=0.24; *P*=.04 (Figure 8). The correlation between RT for Hits in the Whack-the-Mole task and RT for the conjunction search trials of the Visual Search task was not significant, *r*_73_=−0.04; *P*=.74.

## Discussion

### Principal Results

The main objective of this study was to investigate whether the gamified cognitive tasks we developed yield the same patterns of results as their traditional versions. A secondary aim was to determine whether the mode of administration (immersive VR vs desktop VR) and the task administered remotely or in the lab affect performance outcomes.

With regard to the main objective, we found that our gamified tasks replicated the traditional patterns observed in previous studies. Specifically, in the Visual Search task, RTs were shorter for feature search trials than for conjunction search trials, aligning with the established finding that feature searches are overall easier [15]. Additionally, in conjunction search trials, RT increased significantly with display size, a finding known as the display size effect [15,17,18]. Conversely, RTs remained relatively constant across display sizes in feature search trials, demonstrating the well-established pop-out effect [15]. In the Whack-the-Mole task, the overall response inhibition performance aligns with past studies indicating a typical d’ score between 3 and 4, which reflects moderate sensitivity [20,49]. Our participants exhibited false alarms in about 10% of No-Go trials, consistent with the rate reported in the literature [20-22]. Furthermore, in assessing visual short-term memory, our findings showed that our participants had a span between 5 and 7, which is comparable with that reported by studies using the original version of the task [35], as well as those using modern electronic implementations delivered via computers or mobile devices [33].

While replicating the expected pattern of results across the 3 administration conditions, we also found some differences across these conditions. For example, performance in the Visual Search task was faster in the VR-Lab condition by 250 milliseconds than in the Desktop-VR condition. One possibility is that this advantage for immersive VR is due to diminished distraction resulting from blocking out extraneous environmental information when wearing an HMD. However, it could also be due to methodological differences between the 2 administration conditions. Specifically, participants in the Desktop-Lab condition responded by moving and clicking the mouse on the target object. Thus, RT encompassed the time to locate the target, the time to move the mouse, and the time to click. In contrast, in the VR-Lab condition, participants pointed the VR controller to the target object without requiring them to press any button. Thus, RT in this condition involved the time to locate the target and the time to move the controller in 3D space. Perhaps moving the controller in 3D space is performed faster than moving a mouse cursor on a screen. Or perhaps, the difference can be attributed to the need to click the mouse in the desktop condition but not in the VR condition. Notably, we found no difference between the Desktop-Lab and Desktop-Remote conditions, suggesting that differences in the environmental settings (eg, varying screen sizes and distances from the screen at participant homes) and environmental distractors that may be greater at home than the laboratory do not interfere significantly with the assessment of Visual Search abilities.

In the Whack-the-Mole task, performance in the VR-Lab condition was marginally faster by 70 milliseconds than in the Desktop-Lab condition. In contrast to the Visual Search task, in the Whack-the-Mole task, the response requirements were rather similar across the 2 administration conditions. That is, in the VR-Lab condition, participants pulled the trigger of the controller to hit the mole, while in the desktop versions, they clicked the mouse. Thus, taking the results of the 2 tasks together, it seems more likely that in the Whack-the-Mole task, the advantage of immersive VR is due to blocking out access to distracting information from the external environment. Nevertheless, we cannot safely exclude the possibility that the difference is due to subtle differences in the response mode. Perhaps the time needed to pull the trigger on the VR controller is shorter than clicking the mouse button. However, it should be noted that the performance in the Desktop-Lab condition was faster by 160 milliseconds than in the Desktop-Remote condition, despite the fact that the response mode in these 2 conditions was identical. This finding suggests that the differences in overall performance across administration conditions in the Whack-the-Mole task are not methodological.

A possible explanation for the RT differences across the administration conditions of the Whack-the-Mole task is offered by the Perceptual Load Theory. This theory was proposed by Lavie and Tsal [57,58] in the mid-1990s as a potential resolution to the early versus late selection debate in attention and perception. Lavie and Tsal argued that when a task is perceptually demanding (referred to as high load), people dedicate all processing resources to the relevant stimuli early on in the information-processing stream. This results in no interference from distracting irrelevant stimuli, as no spare resources are available to process them. In contrast, when the task is not perceptually demanding (ie, a low-load task), spare resources are available after processing the relevant stimuli. These resources, according to the theory, automatically spill into the processing of irrelevant stimuli. Thus, in low-load tasks, selection takes place late with irrelevant stimuli exerting an influence on performance [59-62]. Notably, in our own research using empirical methods and computational modeling [63], we have shown that inducing a narrow focus of attention may mediate whether distractor effects are present. That is, when the tasks force participants to zoom in their attention to stimuli on the screen, rather than having a more relaxed focus of attention, distracting information can exert no influence even if the task is of low load. Here, the Whack-the-Mole can be more easily classified as low load than high load. More importantly, it can be executed efficiently by maintaining a broader focus of attention, that is, by just focusing attention on a large part in the center of the screen rather than zooming in on a small stimulus. Carrying out the task this way allows for external distractors to slow down performance. If participants’ homes include more environmental distractors than a laboratory, this would translate to more extraneous influence on performance and slowing down of responses. This could also account for the advantage of the VR-Lab condition as donning an HMD serves to effectively block environmental distractors that could influence performance.

A question that arises, though, is why there was no difference between the Desktop-Lab and Desktop-Remote conditions in the Visual Search task? On one hand, the conjunction search trials of the Visual Search task can be thought of as a high-low task that requires a narrow focus of attention, limiting resources to the processing of the relevant stimuli. This would explain why there were no differences across administration conditions. On the other hand, this cannot apply to the feature search trials. Feature search trials are more likely a low load task and, as such, would allow for the processing of distracting information. Yet, no differences were found across administration conditions. Therefore, it seems that further research, in which response modes and other methodological differences are controlled for, is needed to draw more definitive conclusions.

### Comparison With Prior Work

This study examined the impact of administration mode (VR-Lab, Desktop-Lab, and Desktop-Remote) on cognitive performance across 3 tasks: Visual Search, Whack-the-Mole, and Corsi Test. Overall, the results demonstrate that while VR tasks provide a highly immersive and engaging platform for cognitive assessment, the performance differences between VR and traditional desktop-based conditions are task-dependent and vary in magnitude.

Our results from the Visual Search task replicate the well-documented effects of search type and display size [14,15,19], with longer RT observed for conjunction searches than for feature searches and RT increasing with display size in conjunction search trials. Notably, participants in the VR-Lab condition exhibited significantly shorter RTs than those in the Desktop-Lab and Desktop-Remote conditions, suggesting that the immersive nature of VR may enhance visual attention and processing speed in this task. This finding aligns with previous research indicating that VR environments can improve cognitive task performance through increased engagement and spatial immersion [41]. Barrett et al [41] reported longer RT and higher fixation durations in VR compared with 2D environments, which they attributed to the added cognitive demands of immersion. Our results diverge from those of Barrett et al, as the VR-Lab condition here resulted in faster RT, potentially due to the optimized laboratory setup and controlled task parameters. Notably, these patterns of results were obtained with much fewer trials than typically included in the traditional versions of the tasks. For instance, while we ran our gamified Visual Search task with only 96 trials, we produced the same pattern of results as Huang and Pashler [64] and Bravo and Nakayama [65], who ran 1000 and 240 trials, respectively.

The Whack-the-Mole task revealed no significant differences in d’ scores across administration conditions, indicating that accuracy in response inhibition tasks is consistent across VR and desktop modalities. These findings are consistent with those by Bhargava et al [42], who found that VR tasks achieved comparable accuracy to traditional assessments while enhancing engagement. However, RTs for Hits were significantly slower in the Desktop-Remote condition than in both the VR-Lab and Desktop-Lab conditions. This delay may reflect a lack of environmental standardization in remote settings, which has been noted as a limitation of remote cognitive testing [45]. The marginally faster RT in the VR-Lab condition than in the Desktop-Lab condition (*P*=.07) further highlights the potential for VR to enhance cognitive-motor coordination, as observed in other VR-based cognitive tasks [43]. In addition, while Shimi et al [20] and Casey et al [55] had 220 trials and 300 trials, respectively, in the Whack-the-Mole task they administered, we obtained the same results with only 100 trials.

The Corsi Test revealed no significant differences in average span scores across conditions, supporting the conclusion that spatial working memory is not influenced by the mode of administration. This aligns with prior findings that traditional and VR-based versions of the Corsi Test produce comparable results [31,33]. While the observed numeric differences were less and nonsignificant, the slightly lower scores in the Desktop-Remote condition may reflect environmental distractions or variability in participant equipment, which can affect remote testing reliability [45].

The positive correlation between RT for Hits in the Whack-the-Mole task and feature search trials in the Visual Search task suggests an underlying link between response inhibition and attentional processes, particularly in tasks involving rapid response execution. However, the absence of a significant correlation between RT in conjunction search trials and response inhibition suggests that these processes may operate independently under higher cognitive demands. These findings add to the growing literature on individual differences in attentional control [44] and further validate the use of gamified tasks in exploring these relationships.

Our findings emphasize the importance of task design and administration context in cognitive testing. While VR can enhance engagement and, in some cases, improve task performance, the added cognitive load of immersive environments may not universally benefit all cognitive processes [41,45]. The only minor differences in accuracy and spatial memory performance across administration modes highlight the robustness of these tasks to different delivery methods, including remote testing. However, slower RT in remote conditions underscores the need for careful standardization and participant instructions when deploying tasks outside laboratory settings.

Future studies should explore the impact of task complexity and individual differences on performance across VR and traditional modalities. Additionally, longitudinal research is needed to assess whether the observed benefits of VR in certain cognitive domains translate into practical advantages in real-world applications, such as rehabilitation and training.

### Limitations

In this study, we did not assess participants’ prior experience with VR. Previous exposure to VR could have influenced RTs, with participants who have used VR systems before potentially showing faster RTs due to greater familiarity with the technology. This factor should be considered in future studies, and including a questionnaire to assess past VR experience would be a valuable addition to control for this variable and better understand its impact on performance.

While this study focuses on the potential engagement benefits of VR-based cognitive tasks, it is important to acknowledge that VR can also introduce challenges. One key limitation is the potential for cybersickness, eye strain, and general fatigue associated with prolonged VR use. These factors can negatively impact participant performance [66,67] and may have contributed to variability in the results, particularly among participants who were less familiar with VR technology. Although we did not directly assess these issues by asking participants, no participants in the VR group reported any difficulties or discomfort with VR during or after their participation. While this suggests that cybersickness or fatigue was not a major confound, future studies could benefit from incorporating standardized self-report questionnaires or physiological measures to better isolate the impact of VR engagement on cognitive performance.

Notably, we did not include a paper-and-pencil comparison group, as our primary aim was to contrast the gamified VR tasks with traditional desktop-based digital assessments. Including a paper-and-pencil comparison could provide insights to cross-modality generalizability, but it would also introduce additional variables unrelated to our core research objective, such as differences in response modalities and environmental influences. Nevertheless, we acknowledge that such a comparison could be valuable in future research to examine broader ecological validity, recognizing that certain tasks in our study (eg, Whack-the-Mole) are not feasible in a paper-based format.

### Conclusions

This study demonstrated that gamified VR tasks can replicate well-established cognitive performance patterns using fewer trials, highlighting their potential as efficient and engaging tools for assessing attention, response inhibition, and visual short-term memory. By comparing performance across immersive VR, desktop-based, and remote administration conditions, we found that VR-based tasks maintained ecological validity and produced results consistent with traditional methods, suggesting that increased immersion does not compromise data quality. These advantages are particularly relevant for reducing participant fatigue and enhancing motivation, factors essential for high-quality cognitive assessment.

At the same time, VR introduces methodological variables that require further attention. In this study, we did not directly assess cybersickness, fatigue, or prior VR experience, which may influence individual performance. In addition, comparisons to paper-and-pencil tasks were not included, as the tasks examined here are typically administered in computerized form. Future research should explore these aspects systematically and investigate how task-specific features and environmental settings interact with immersion to affect cognitive outcomes. Overall, our findings support the use of immersive VR as a viable and scalable platform for cognitive assessment, especially in applications that benefit from high engagement and ecological realism.

## Supplementary material

10.2196/65836 Multimedia Appendix 1 Example of a feature search trial used in the Visual Search task of this experimental study. In this trial, participants searched for a gray 2-sided axe (target) among 24 brown 2-sided axes (distractors). This is a trial with a display size of 25. The red outline is added for illustration purposes and was not present in the actual display viewed by participants.

10.2196/65836 Multimedia Appendix 2 Mean span scores in the Corsi block-tapping task across the 3 administration conditions. Span scores reflect the longest sequence of targets correctly recalled by participants. Error bars represent 95% confidence intervals.

## Abbreviations

ACE-III: Addenbrooke’s Cognitive Examination—III
HMD: head-mounted display
RT: reaction time
VR: virtual reality
