# Supplementary figures and images for: Virtual Reality Gamification of Visual Search, Response Inhibition, and Visual Short-Term Memory Tasks for Cognitive Assessment: Experimental Study
Source: JMIR Form Res. 2025 Jul 29;9:e65836. doi: 10.2196/65836 (PMC12306913; doi:10.2196/65836)

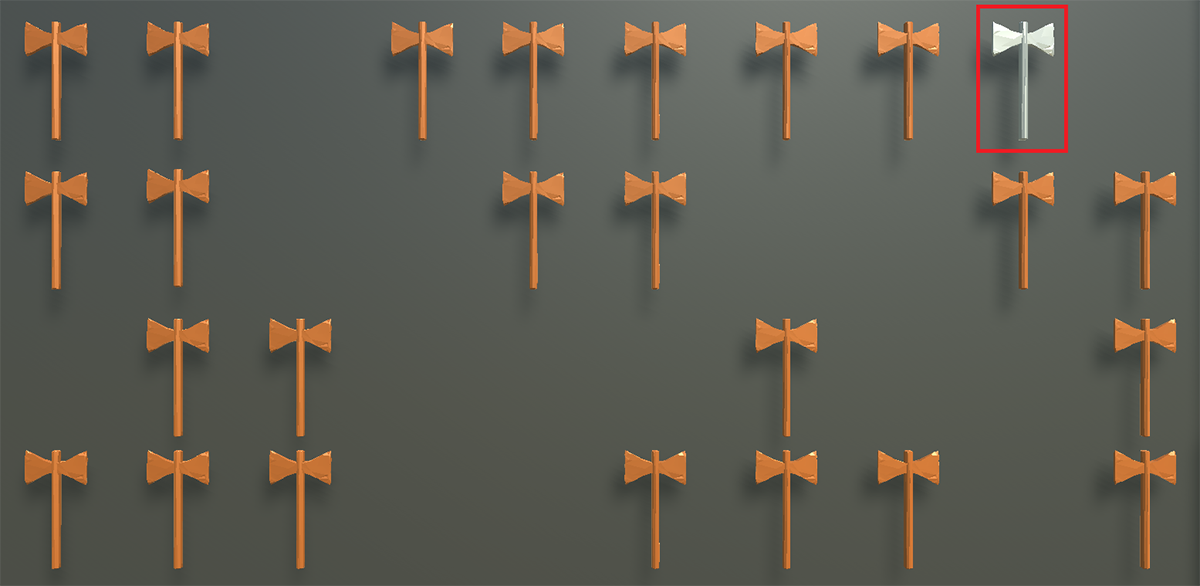

Supplement: Multimedia Appendix 1 [file formative-v9-e65836-s001.png]

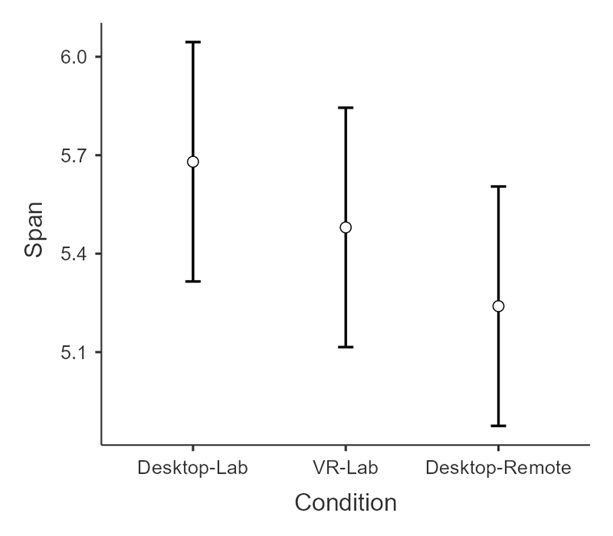

Supplement: Multimedia Appendix 2 [file formative-v9-e65836-s002.png]
